# Epizoochorous dispersal by ungulates depends on fur, grooming and social interactions

**DOI:** 10.1002/ece3.3768

**Published:** 2018-01-03

**Authors:** Océane Liehrmann, Flore Jégoux, Marie‐Alice Guilbert, Francis Isselin‐Nondedeu, Sonia Saïd, Yann Locatelli, Christophe Baltzinger

**Affiliations:** ^1^ Irstea Centre de Nogent‐sur‐Vernisson Unité de Recherche Écosystèmes Forestiers Nogent‐sur‐Vernisson France; ^2^ Direction Recherche et Expertise Unité Ongulés sauvages ONCFS Birieux France; ^3^ Département d'Aménagement et d'Environnement UMR 7324 CITERES École Polytechnique de l'Université François Rabelais Tours France; ^4^ Réserve Zoologique de la Haute Touche MNHN Obterre France

**Keywords:** behavior, *Capra aegagrus hircus*, *Cervus elaphus*, epizoochory, *Equus asinus*, seed dispersal distance, seed retention time, *Xanthium strumarium* L.

## Abstract

The transport phase of the animal‐mediated plant dispersal process is critical to dispersal effectiveness as it determines the spatial distribution of the diaspores released and their chance for further recruitment. Assessing this specific phase of the dispersal process generally requires combining diaspore retention times with the associated distances covered. Here, we specifically tested the effect of grooming behavior, interindividual contacts and ungulate fur on diaspore retention times and associated dispersal distances for the hooked diaspores of *Xanthium strumarium* L. experimentally attached to tamed individuals of three ungulate species. We used a comparative approach based on differing fur quality on different body zones of these three ungulates. During 6‐hr sessions, we monitored for grooming and social interactions that may induce intended or inadvertent diaspore detachment. Additionally, we proposed innovative approaches to directly assessing diaspore dispersal distances by red deer in situ. Fat‐tailed functions fitted diaspore retention time, highlighting the potential for long‐distance dispersal events. The longer the hair, the higher the retention capacity of diaspores in the animal's fur. As predicted, donkey retained diaspores longer than red deer and dwarf goat; and we also confirmed that diaspores attached to the short hair of the head fell off more quickly than did those on the other body zones. Dwarf goat groomed more often than both red deer and donkey, but also when it carried diaspores. Up to 14% of the diaspores detached from animal fur after specific grooming behavior. We observed, in controlled conditions, for the first time and for each ungulate species, interindividual transfers of diaspores, representing 5% of the diaspores attached to animals’ fur. Our results militate for incorporating animal behavior into plant dispersal modeling approaches.

## 1 INTRODUCTION

Seed dispersal is a key process for the dynamics of plant populations, particularly in a transient and fast‐changing environment (Bullock, 2012). Current plant populations have to face multiple threats linked to anthropic modifications of both biotic and abiotic conditions: introduction of invasive species (Chuong et al., 2016; Constible, Sweitzer, Vuren, Schuyler, & Knapp, 2005), climate change, habitat alteration, and landscape fragmentation (McConkey et al., 2012). Also, rare events of long‐distance seed dispersal can explain the current distribution of certain plants (Cain, Milligan, & Strand, 2000; Higgins & Richardson, 1999). These events can be linked to climatic hazards (tornadoes, hurricanes …), biotic accidents (mud attached to animal legs, ingestion and defecation of viable seeds, human activities) or other abiotic forms of transport (hydrochory). However, according to Bertrand et al. (2011), lowland plant communities are composed of species with great phenotypic plasticity for local persistence as temperature increases and their composition seems to remain the same, indicating reduced turnover and low dynamics of emigration/immigration.

Many plants, both native and exotic (Eschtruth & Battles, 2009), interact with animal vectors for their dispersal, via endo‐ and epizoochory (Albert, Auffret, et al., 2015; Albert, Mårell, Picard, & Baltzinger, 2015). In temperate areas, both wild and domestic ungulates (Bartuszevige & Endress, 2008; Whitacre & Call, 2006) are involved in the long‐distance dispersal of many plants. For instance, red and roe deer facilitated the expansion of a rare toxic plant, *Cynoglossum germanicum* (Boulanger et al., 2011), and thus affected its spatial distribution over a 30‐year period. Sheep promoted the colonization of grassland species on newly created bare soil via epizoochory (Freund, Eichberg, Retta, & Schwabe, 2014); Rico, Boehmer, and Wagner (2014); Rico, Holderegger, Boehmer, and Wagner (2014) also demonstrated that rotational shepherding improved landscape genetic connectivity in temperate grasslands. As such, large ungulates can promote fluxes and functional connectivity among plant populations and associated habitats, and allow the colonization of new habitats. This leads to consider them as dynamic ecological corridors (Couvreur, Christiaen, Verheyen, & Hermy, 2004) and potential tools for the restoration of degraded habitats. These dispersal effects can further cascade at the community level and contribute to the dynamics of plant metacommunities.

We generally distinguish three phases for the study of zoochory: the initiation, when the animal loads the seed; the seed transport, corresponding to the trajectory covered; and the establishment, when and where the seed is released. The distance and effectiveness of animal‐mediated dispersal are determined by the combined effect of the time seeds are retained by the vectors, their associated movements and the ability of the seeds to germinate and produce an adult (Nathan et al., 2008; Schupp, 1993 and Schupp, Jordano, & Gómez, 2010). To build seed dispersal kernels, seed retention time by animals is assessed ex situ under controlled conditions (Picard et al., 2015), then combined with the movements of wild animals obtained in situ by telemetry (VHF radio transmitter or GPS) or through direct observation (Mårell, Ball, & Hofgaard, 2002). Specifically for large ungulates, real‐time monitoring of seed dispersal distances is made complex due to the magnitude of the movements in areas with more or less visibility (grasslands vs. forests) and the small seed size.

A recent European meta‐analysis by Albert, Auffret et al. (2015) indicated that 44% of the regional flora was dispersed by wild and domestic ungulates, and more specifically, they showed that fur‐epizoochory was the strongest ecological filter (Myers & Harms, 2009) when compared to hoof‐epizoochory and endozoochory. Fischer, Poschlod, and Beinlich (1996) and Albert, Mårell et al. (2015) highlighted that plants taller than 20 cm were mostly dispersed via epizoochory due to a higher encounter rate between animal flanks and infructescence. Elongated seeds, or seeds bearing elongated appendages or hooks, promote epizoochory (Couvreur, Verheyen, & Hermy, 2005). However, previous studies have shown that epizoochory also depends on fur quality. Some *Daucus carota* seeds were retained more than 166 days in sheep *Ovis aries* wool (Manzano & Malo, 2006) whereas *Triglochin palustris* seeds remained roughly 3 hr in the fur of fallow deer (Kiviniemi, 1996). Kulbaba, Tardif, and Staniforth (2009) linked seed morphology and different kinds of fur, using a gradient from mouse to moose. They found a positive relationship between hair length, fur density, and seed adherence for *Bidens frondosa* and *Xanthium strumarium*.

Indeed, fur quality depends on animal species but also differs among body parts on a single individual. Hair length or density is different on the legs, the head, or the flanks. de Pablos and Peco (2007) found that seeds are better attached to the flanks than to the legs of cattle and sheep. Further, fur quality varies seasonally, with thicker coats in winter than in summer and with hair loss during the molt (e.g., in Alpine ibex *Capra ibex ibex*, Couturier, 1962). These fur differences can affect the diversity and quantity of the plants and diaspores transported by animals. Picard and Baltzinger (2012) found a higher diversity of plants and larger quantities of seeds in wild boar fur than in red and roe deer fur. These fur quality differences can also influence the animal's sensitivity to foreign objects trapped in their fur and promote grooming behavior, both intraspecific cleaning behavior and exo‐scratching behavior (Sarasa et al., 2011; Sorensen, 1986) to detach diaspores. Some other behaviors, such as mud baths taken by wild boar or elephant to reduce their parasitic load (Vanschoenwinkel et al., 2008, 2011), could also affect the attachment capacity of their fur. However, the effect of grooming behavior on diaspore retention has rarely been included in seed dispersal models because its monitoring is highly time consuming (Couvreur et al., 2005).

In this study, we experimentally assessed the effect of fur quality and grooming behavior on retention time and associated dispersal distances of the large hooked diaspores of the common cocklebur *X. strumarium*. We used a double comparative, intra‐ and interspecific approach based on different fur quality on three body zones (head, flanks, and rump) of tamed individuals of three ungulate species: red deer *Cervus elaphus*, Poitou donkey *Equus asinus,* and dwarf goat *Capra aegagrus hircus*. Through continuous monitoring, we observed the social interactions and grooming behavior that may induce diaspore detachment. We had two main goals in this study on epizoochorous diaspore dispersal by large ungulates. First, we experimentally assessed diaspore retention time functions and how both fur quality and grooming behavior affect these functions. Second, we assessed diaspore dispersal distances through complementary approaches, with red deer as the model vector.

Concerning diaspore retention time assessment, Bullock et al. (2011) tested different functions in a review of datasets covering various time periods, and animal and plant species. They found the simple exponential function to be the best fit for a short monitoring time (<48 hr), whereas fat‐tailed functions best fit datasets obtained over longer periods. We fitted these two types of functions (Table 1) to our diaspore retention time datasets. According to Bullock et al.'s findings and consistent with the short monitoring period chosen, we expected the simple exponential function to provide the best fit. We then tested how ungulate fur quality influenced diaspore retention capacity and retention time. At the interspecific scale, we predicted more diaspores to be retained longer in the long thin hair of Poitou donkey than in the thicker wavier hair of red deer, which in turn should retain more diaspores than in the shorter wavy hair of dwarf goat. At the intraspecific scale and for each animal species, we predicted that diaspores would remain longer in the longer hair on the rump and the flanks than in the shorter hair covering the animal's head (Table 2). Finally, we tested whether the presence of diaspores in the animal's fur provoked specific grooming behavior (Sorensen, 1986). We initially predicted that individuals bearing diaspores would groom more frequently than individuals without diaspores (stimulus‐driven‐grooming hypothesis, Hart, Hart, Mooring, & Olubayo, 1992), and secondly, that small‐bodied animals, for an equal diaspore load, would be more involved in auto‐grooming behavior than larger ones (body‐size principle and programmed‐driven‐grooming hypothesis, Hart et al., 1992; Mooring, Benjamin, Harte, & Herzog, 2000).

**Table 1:** Functions used to fit diaspore retention time or dispersal distance: *p* corresponds to the proportion of diaspores left on the animal vector; *t* represents time (*d* replaces *t* for distance); *a* and *b* figure parameters to be estimated (Bullock et al., 2011; Wichmann et al., 2009)

Function	Formula
Simple exponential	*p*(*t*) = *a*.exp^(−*bt*)^
Power exponential	*p*(*t*) = *a*.exp(−*t* ^*b*^)
Double exponential	*p*(*t*) = *a*.exp(exp^(−*bt*)^)
Inverse power	*p*(*t*) = *a*.t^−*b*^

**Table 2:** Winter fur characteristics of three body zones (head, flanks, and rump) of our three animal vectors (dwarf goat, red deer, and Poitou donkey). We measured fur depth (perpendicular to animal body) and hair length of all individuals used in the experiment at three random points on each body zone, qualitatively estimated hair thickness from low (+) to high (+++) and described hair curliness

Animal vector	Body zone	Hair curliness	Fur depth (mm)	Hair length (mm)	Hair thickness
Dwarf goat *Capra aegagrus hircus*	Head	Smooth	14.0 ± 5.9	22.0 ± 4.1	+
	Flanks	Micro wavy	23.0 ± 4.4	46.0 ± 6.5	++
	Rump	Micro wavy	31.0 ± 4.7	62.0 ± 7.4	++
Red deer *Cervus elaphus*	Head	Smooth	9.0 ± 1.3	15.0 ± 2.5	+
	Flanks	Micro wavy	24.0 ± 1.8	54.0 ± 2.8	+++
	Rump	Micro wavy	29.4 ± 3.5	65.0 ± 5.0	+++
Poitou donkey *Equus asinus*	Head	Curly	36.0 ± 4.8	73.0 ± 6.0	+
	Flanks	Wavy and dreadlocks	28.0 ± 1.4	161.0 ± 22.8	+
	Rump	Wavy	28.0 ± 4.0	124.0 ± 37.2	+

Concerning diaspore dispersal distance assessment, we benefitted from three different approaches. In the first experimental approach, we directly retrieved dispersal distances through live monitoring of diaspores experimentally attached to tamed red deer retained in a fenced wooded pen and equipped with GPS collars. In the second classical approach, we coupled experimentally assessed diaspore retention time with red deer movements under natural conditions. Then, we imagined a third innovative approach, where we implemented live dispersal monitoring of diaspores attached to wild red deer equipped with GPS/GSM collars under natural conditions.

## 2 MATERIAL AND METHODS

### 2.1 Study sites, animals and diaspores

The main diaspore retention experiment was conducted from 22 February 2016 to 11 March 2016 with both domestic and tamed animals from Réserve Zoologique de la Haute Touche (46.885167°N, 1.076445°E; hereafter RZHT) situated in the Parc naturel régional de la Brenne (Obterre, France). We manipulated six individuals, mainly females, from each animal species: red deer, Poitou donkey, and dwarf goat (five females and one male). For a given animal species, all six individuals occupied a specific wooded pen, ranging from 0.68 to 1.81 ha. We built our experiment on the differences both in body size and fur quality of these three large ungulates to test the effect of fur quality and grooming behavior on diaspore retention time (Table 1).

In addition to this site, we worked in the Domaine National de Chambord (47.616155°N, 1.517261°E; hereafter DNC, Chambord, France) under the “Programme de la Fondation François Sommer” led by the National Hunting & Wildlife Agency. In DNC, the red deer population comprises about 700 individuals contained in a 5400‐ha wall‐fenced park. We monitored native red deer hinds from the DNC, trapped during capture–mark–recapture events in 2015 (*n* = 6) and in 2016 (*n* = 5). The captured individuals were equipped with GPS collars (WildCell SG Collar SOB/GSM) that allowed us to track their movements and quantify the distances they covered during different time slots.

All experiments from RZHT were performed at the occasion of medical training for routine morphological veterinary examinations. Experiments complied with the ethical standards of animal manipulation as defined by the French laws on animal welfare (Décret n°2013‐118, license number 36‐145‐002 for RZHT & 2014178‐0009 for DNC).

The large diaspores of the common cocklebur *X. strumarium* are well‐adapted to epizoochorous transport (bearing more than 50 hooks and releasing diaspores at 0.75 m above ground, Kleyer et al., 2008) and have been used in previous studies on external diaspore retention time (Couvreur et al., 2005; Kiviniemi & Telenius, 1998 and Kulbaba et al., 2009). We collected diaspores during diaspore shedding period in fall 2015 (Loiret, France) in situ along the banks of the Loire River where common cocklebur is considered to be an invasive plant. We then sterilized each diaspore for 70 s at 800 W in a microwave oven. The large size of the diaspores, particularly those used in this study (length 28.90 ± 0.06 mm; width 18.20 ± 0.85 mm; weight = 400.00 ± 0.06 mg), facilitated monitoring from a convenient distance without disturbing the animals, although the large size was also likely to generate specific grooming behaviors (Sorensen, 1986).

### 2.2 Diaspore retention time experimental design

**Figure 1:**
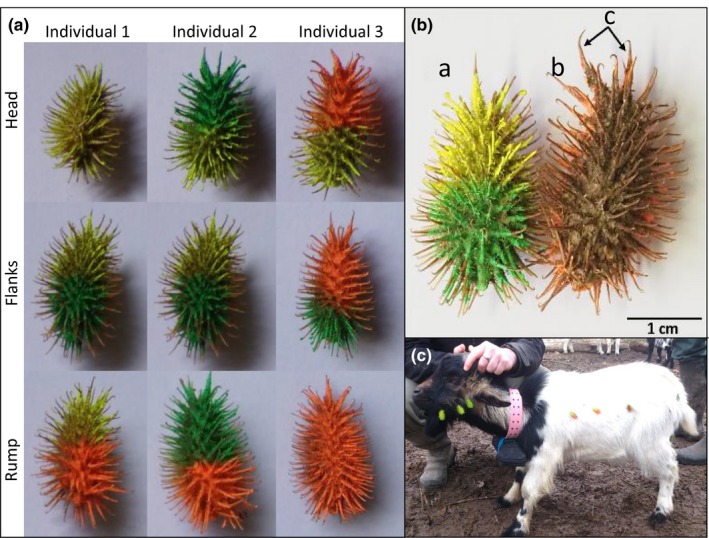
A—Bicolor code used for the identification of both individuals and body zones. B—Cocklebur *Xanthium strumarium* (a) painted side, (b) unpainted side attached to the hairs, and (c) two large spikes indicating the distal part of the diaspore. C—Left side of a dwarf goat bearing painted cockleburs *X. strumarium* showing the pink collar equipped with the hiking GPS

For each animal species, we monitored three to four individuals per day during 6‐hr scanning sessions (from 9:00 until 15:00) for 5 days (dwarf goat from 22 February 2016 to 26 February 2016, red deer from 29 February 2016 to 4 March 2016 and donkey from 7 March 2016 to 12 March 2016). Each individual was monitored by a single operator. We manually attached 18 diaspores per animal onto two to three individuals equipped with a hiking GPS (Garmin Gpsmap 62s) attached to plastic collar (Figure 1c) and which had been programmed to record one location per minute. One to two control individuals received no diaspores. The fluorescent painted half of each diaspore identified the individual and its associated body zone according to a bicolor code based on three different colors (yellow, orange, and green). For instance, the green individual 2 (Figure 1a) had the distal part of each diaspore painted in green, while a different color for the proximal part corresponded to a specific body zone. The nonpainted half of each diaspore was manually attached to the animal fur without any modification of its sticking ability (Figure 1b).

We attached the diaspores as follows, three on each side of three body zones (head, flank, and rump, Figure 1c) where diaspores can naturally cling to animal fur, in agreement with cocklebur diaspore releasing height and accidental attachment to the head when grazing neighboring plants (Kiviniemi, 1996). In total, we monitored 13 times red deer, 14 times donkey, and 15 times dwarf goat, corresponding to 234, 252, and 270 diaspores, respectively. Each individual has been monitored from one to three times. We continuously monitored the half‐painted cocklebur diaspores through binoculars and telescopes (magnifying up to 60 times) to identify the cause, location, and moment of detachment.

We monitored the presence of each diaspore from the beginning at 1, 5, 10, and 15 min and then regularly every 15 min until the end of each 6‐hr scanning session. If one of the operators observed a diaspore as it detached, they noted the cause, the precise location on a paper map, and the time of detachment. After each daily 6‐hr scanning session, we entered the wooded pen to remove the diaspores still clinging to the animals but also to retrieve and assign each diaspore that had fallen on the ground to one of the monitored individuals, thanks to the color code. We also continuously and simultaneously tracked the behavior of individuals bearing diaspores and their paired controls, without diaspores. Each operator noted any behavior that could cause a diaspore detachment from the animal. We differentiated grooming behaviors (auto‐grooming vs. allo‐grooming, self‐shaking, rolling, and scratching) from other behaviors that could inadvertently cause diaspore detachment (playing time, agonistic interactions, mounting, and lying down) (see associated ethogram, Table 3).

**Table 3:** Ethogram listing behaviors that could induce intended (grooming) or inadvertent diaspore detachment from animal fur

Behavior associated with intended diaspore detachment from animal fur (grooming)	
Auto‐grooming	An individual grooms or scratches itself with its mouth, legs, or horns (dwarf goat)
Allo‐grooming	An individual grooms a conspecific
Scratching	An individual scratches itself against an object (tree, fence, hut, …)
Rolling	An individual rolls itself on the ground
Self‐shaking	An individual shakes its body or its head

Behavior associated with inadvertent diaspore detachment from animal fur	
Social interactions	
Games	An individual chases a conspecific or simulates fight
Mounting	An individual simulates a reproductive behavior
Agonistic Interactions	An individual keeps a conspecific away (bite, attack, threat) resulting in a sudden movement
Head rest	An individual puts its head on a conspecific
Other activities	
Rear	An individual stands on its posterior legs (usually to get leaves in trees)
Lying	An individual lies on the ground to rest or ruminate

### 2.3 Diaspore dispersal distance experimental design

For the experimental approach, we retrieved the total distance walked by each red deer up to the moment when a diaspore detached from the animal. For the classical approach, we coupled diaspore retention time with red deer movements. We used GPS locations scheduled every 2 hr over 1 year and retrieved from the six individuals equipped in 2015 (WildCell SG Collar SOB/GSM). We measured the Euclidian distances (Jégoux, 2016; Pellerin, Picard, Saïd, Baubet, & Baltzinger, 2016) walked during 2 hr between 9:00 and 11:00 in February and March 2015 and also during a six‐hour period from 9:00 to 15:00 corresponding to the diaspore retention time in the experimental design. We then combined the proportion of remaining attached diaspores with the distance traveled during the same amount of time. For the innovative live‐monitoring approach, five new individuals were trapped and equipped with similar GPS collars in 2016 (from 12 January 2016 to 23 February 2016). We took this capture opportunity to attach 40 painted common cocklebur diaspores, 20 on the chest and 20 on the rump of each individual caught. We specifically programmed the GPS collars to retrieve the animal location every 5 min for the first three hours after their release. We were thus able to quickly back track the trajectory of each individual in the field using the first GPS locations, transmitted via GSM, to try to recover some of the detached diaspores lying on the ground.

### 2.4 Diaspore retention time/dispersal distance functions and fur quality effects

We modeled the cocklebur diaspore retention time for the three species with the four functions tested by Bullock et al. (2011) and summarized in Table 1. We used the nlme package to adjust nonlinear mixed‐effects models with the proportion of remaining diaspores as the response variable. We first defined animal species as a fixed effect to test for interspecific differences and then body zone to test for the effect of intraspecific fur variability for each animal species. We accounted for individual variability by setting individual within date as a random effect.

We used the same method to model the cocklebur diaspore dispersal distance for tamed red deer and took into account the effect of body zone. We used the total distances traveled by the animals, as a maximal distance covered, provided by the data collected from the hiking GPSs attached to the plastic collar; to avoid the bias linked to the size of the wooded pen, we did not use the Euclidian distances between the fallen diaspores and the animals’ place of release in the experimental approach.

We selected the best function according to AIC and then retrieved the associated fitted parameters (*a* and *b*, Tables 1 and 4). We also derived half‐life diaspore retention time (i.e., time corresponding to 50% diaspore loss) from the fitted functions. We tested the effect of fur morphology (Table 2, using hair length or fur depth separately, as they were correlated for deer, *r*² = .86, *p* < .001, and dwarf goat, *r*² = .86, *p* < .001) on diaspore retention capacity. We used the lme4 package to fit generalized linear mixed‐effects models with diaspore fate (absent or still present after 6 hr, binomial distribution) as the response variable, hair length or fur depth as dependent variables, and body zone within individual within date as a random effect to account for our experimental design. Differences among animal species and among body zones were estimated using Tukey contrasts for multiple comparisons of means.

**Table 4:** AIC values of the four different functions (Table 1) fitted to (i) diaspore retention time datasets at the inter‐ and intraspecific (body zones) scale and (ii) diaspore dispersal distance dataset at the intraspecific (body zones) scale for *Cervus elaphus*. AIC in bold corresponds to the selected function, for which *a* and *b* parameters are indicated. Values of *a* (and *b*) sharing different upper letters indicate significant differences among species or body zones. Half‐life retention time (min) and dispersal distance (m) are indicated at the intraspecific scale

(i) Retention time	Simple exponential	Power exponential	Double exponential	Inverse power	Fitted parameter *a*	Fitted parameter *b*	Half‐life retention time (min)
Interspecific scale	−1,887.06	−**2**,**184.14**	−1,704.25	−1,779.45			
*** **Capra aegagrus h*.					1.756^α^ ± 0.131	0.126^α^ ± 0.003	
*** **Cervus elaphus*					1.730^α^ ± 0.127	0.095^β^ ± 0.003	
*** **Equus asinus*					2.638^β^ ± 0.091	0.011^γ^ ± 0.002	
Intraspecific scale							
* Capra aegagrus h*.	−**203.52**	−176.42	−124.28	8.76			
Head					0.652^α^ ± 0.035	0.019^α^ ± 0.002	14
Flanks					0.707^α^ ± 0.031	0.006^β^ ± 5e‐04	61
Rump					0.805^β^ ± 0.050	0.004^γ^ ± 2e‐04	132
*** **Cervus elaphus*	−682.80	−**812.34**	−562.15	−598.33			
Head					1.459^α^ ± 0.058	0.182^α^ ± 0.007	1
Flanks					1.668^β^ ± 0.057	0.083^β^ ± 0.005	9
Rump					2.013^γ^ ± 0.135	0.055^γ^ ± 0.003	>360
*** **Equus asinus*	−**1**,**884.29**	−1,874.32	−1,858.45	−1,871.06			
Head					0.930^α^ ± 0.013	5e‐04^α^ ± 8e‐05	>360
Flanks					0.977^β^ ± 0.013	5e‐05^β^ ± 7e‐05	>360
Rump					0.966^β^ ± 0.016	6e‐05^β^ ± 5e‐05	>360

(ii) Dispersal distance	Simple exponential	Power exponential	Doubleexponential	Inverse power	Fitted parameter *a*	Fitted parameter *b*	Half‐life dispersal distance (m)
*Cervus elaphus*	−**477.12**	−369.82	−407.51	−278.78			
Head					0.842^α^ ± 0.037	0.0022^α^ ± 2e‐04	237
Flanks					0.700^β^ ± 0.029	0.0006^β^ ± 5e‐05	561
Rump					0.814^α^ ± 0.071	0.0002^γ^ ± 2e‐05	2,437

### 2.5 Diaspore retention and animal behavior

We first tested whether the presence of attached diaspores increased grooming behavior and self‐shaking events at the individual scale (all three body zones combined). We used nonparametric paired Wilcoxon tests for each animal species to compare grooming occurrence between paired individuals with diaspores and controls without diaspores that were monitored by the same operator on a given day. We then tested whether body size influenced grooming events by comparing grooming occurrence among our three animal species using a Kruskal–Wallis nonparametric test, followed by a post hoc Dunn test for multiple comparisons.

We also tested whether the proportion of diaspores detached by grooming differed among animal species. We used the lme4 package to fit a generalized linear mixed‐effects model, with diaspore detached by grooming as the response variable (yes or no, binomial distribution) and species as the fixed effect, and we again accounted for individual variability by setting individual within date as a random effect.

Using GIS (ArcGIS, ESRI 2011), we also assessed the proportion of the diaspores retrieved in the wooded pen and those found in the vicinity (within 2 m) of trees, huts, or fences and for which detachment could be attributed to a rubbing behavior.

All statistical analyses were performed within the R environment (R Core Team 2015).

## 3 RESULTS

### 3.1 Diaspore retention time and monitoring length

**Figure 2:**
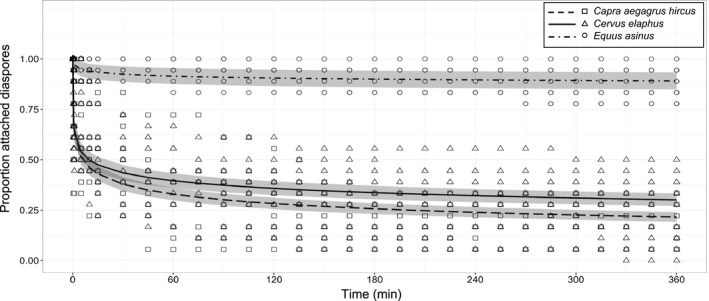
Power exponential functions fitted for the proportion attached diaspores over time (min) at the interspecific scale: *Capra aegagrus hircus* (open square, regular dashed line), *Cervus elaphus* (open triangle, continuous line), and *Equus asinus* (open circle, irregular dashed line). Gray bands correspond to modeled standard errors

**Figure 3:**
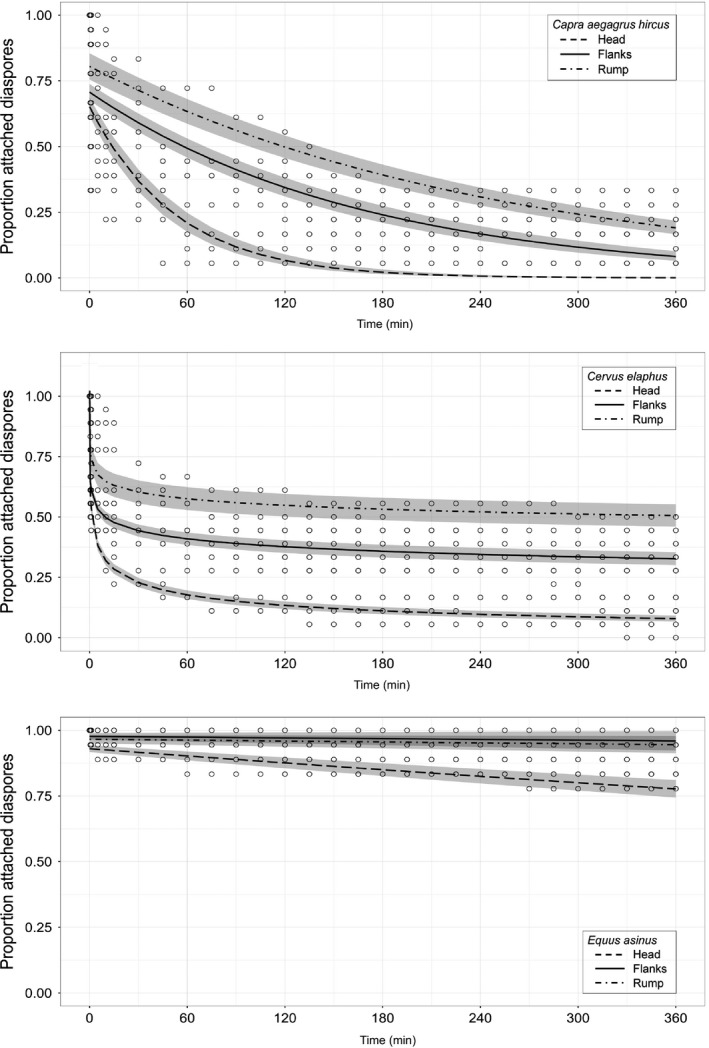
Selected functions fitted for the proportion attached diaspores over time (min) at the intraspecific scale (body zones): top box for *Capra aegagrus hircus,* middle box for *Cervus elaphus,* and bottom box for *Equus asinus;* within boxes, head (regular dashed line), flanks (continuous line), and rump (irregular dashed line). Gray bands correspond to modeled standard errors

At the interspecific scale, we found the power exponential function (Table 4, Figure 2) to best fit the proportion of diaspores remaining on the animal over time. At the intraspecific scale (body zone), we again kept the same function for red deer; however, the simple exponential function better fitted both dwarf goat and donkey data (Table 4, Figure 3).

### 3.2 Effects of fur quality

The longer the hair (*r*² = 0.64, *Z* = 9.21, *p* < .001), or the deeper the fur (*r*² = 0.54, *Z* = −5.49, *p* < .001), the higher the diaspore retention capacity on the animals after 6 hr. At the interspecific scale, the functions that best fitted to each animal species significantly differed from one another. The proportion of diaspores remaining on the animals over time was significantly higher for donkey than for red deer than for dwarf goat (Table 4, Figure 2). At the end of the 6‐hr monitoring sessions, donkey still bore 88% of the attached diaspores, significantly more than red deer (26%; *Z* = 9.21, *p* < .001) or dwarf goat (16%; *Z* = 10.27, *p* < .001). When comparing body zones, significantly more diaspores fell from the head than from the flanks (*Z* = 5.27, *p* < .001) or the rump (*Z* = 7.39, *p* < .001). The diaspores remained even longer on the rump than on the flanks for both dwarf goat and red deer, but not for donkey (Table 4, Figure 3). Half‐life diaspore retention times varied among and within animal species (see Table 4 for detailed results).

### 3.3 Effects of grooming behavior

**Figure 4:**
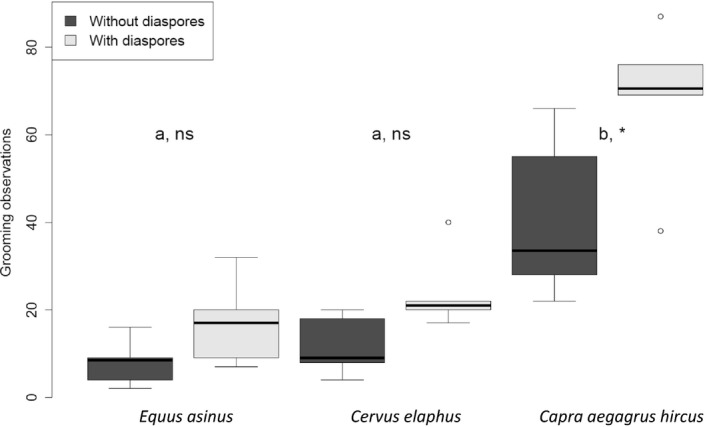
Box plots representing the grooming observations per animal species (*Equus asinus*,* Cervus elaphus*, and *Capra aegagrus hircus)* for individuals with diaspores (light gray) or without (dark gray). Different letters (a, b) indicate significant differences between animal species. For each species, * indicates a significant difference at *p* < .05 and ns a nonsignificant difference, whether the individuals carried diaspores or not

Dwarf goats groomed significantly more when they carried diaspores (*W*_(5)_ = 21, *p* = .028). We observed a similar but nonsignificant trend for donkey and red deer (Figure 4). Dwarf goat groomed also significantly more than did red deer (*Z* = 4.41, *p* < .001) or donkey (*Z* = 4.43, *p* < .001), even in the absence of attached diaspores; however, there were no differences between red deer and donkey. At least 7% of the detached diaspores were detached from the dwarf goat's fur following specific grooming behaviors (seven diaspores by self‐shaking and six by scratching), 10% from donkey (one diaspore by scratching and two by other grooming behaviors), and up to 14% from red deer (13 diaspores by self‐shaking and 14 by scratching). But this proportion did not depend on animal species (*χ*²_(2)_=2.95, *p* = .23). We recovered more detached diaspores in the vicinity of trees, huts, or fences; up to 31% for dwarf goat (25 head‐, 25 flanks‐, and 10 rump‐diaspores), 14% for red deer (12 head‐, 8 flanks‐, and 8 rump‐diaspores) and only 3% for donkey (one single head‐diaspore).

### 3.4 Diaspore dispersal distance

**Figure 5:**
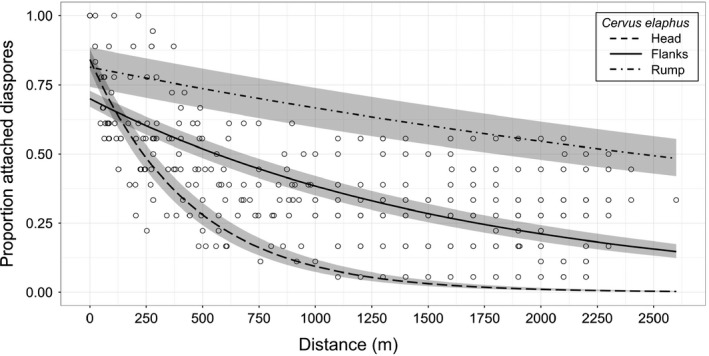
Simple exponential functions fitted for the proportion attached diaspores over distance (m) for *Cervus elaphus:* head (regular dashed line), flanks (continuous line), and rump (irregular dashed line). Gray bands correspond to modeled standard errors

Contrary to diaspore retention time, the simple exponential function was retained to explain overall diaspore dispersal distance for red deer (Table 4, Figure 5) in our experimental approach. In February–March 2015, red deer had covered on average 342 ± 330 m (max. 2260 m) in the first 2 hr after release, from 9:00 to 11:00 in the DNC; this corresponds to the time slot when most of the diaspores detached from the fur (85% from the head, 60% from the flanks and 40% from the rump). If we consider the total 6‐hr slot from 9:00 to 15:00 corresponding to our diaspore retention experiments, distances covered reached on average 423 ± 387 m (max. 2,400 m) and 5% more of the diaspores were detached from each body zone. This classical approach would have better matched detachment kinetics had more frequent locations been taken. Finally, our innovative approach highlighted that most (>70%) of the painted cocklebur diaspores that we recovered came from the chest of the individuals and were retrieved within 20 m. However, we also found one diaspore 2.85 km away (Table 5), 50 min after the animal had been released.

**Table 5:** Results from our innovative approach to assessing diaspore dispersal distance in situ at the Domaine National de Chambord. Average dispersal distance corresponds to the average distance between where diaspores were recovered and where the red deer individual had been released

Capture date	Individual	Diaspores recovered (%)	Recovered diaspores from the chest	Recovered diaspores from the rump	Dispersal distance average–maximum (m)
01/12/2016	#1	55	17/20	5/20	13.12–41.24
01/26/2016	#2	48	17/20	2/20	256.38–2,846.78
02/09/2016	#3	5	2/20	0/20	14.01–17.06
02/23/2016	#4	25	7/20	3/20	9.57–9.57
02/23/2016	#5	35	13/20	1/20	20.26–43.2

### 3.5 Diaspore transfers

During the diaspore retention time experiment, we have observed real‐time diaspore transfers from one individual to another conspecific for all three animal vector species: twice for red deer, seven times for dwarf goat, and up to 29 times for donkey.

## 4 DISCUSSION

### 4.1 Fur quality and diaspore retention time

Our study demonstrates how fur quality affects diaspore retention dynamics thanks to a coupled inter‐ and intraspecific approach with domestic and tamed wild ungulates. Few studies (Kiviniemi, 1996) have tested the variable fur qualities on different body parts of the same individual. This intraspecific approach has the advantage of controlling for external confounding effects. At the interspecific scale, the power exponential function was the best fit to describe diaspore retention time among deer, dwarf goat, and donkey. If we refer to Bullock et al. (2011) for diaspores with or without specific adaptation to epizoochory, this was not expected for a short monitoring length. In fact, the authors found that the simple exponential model gave the best fit to the data from shorter periods (<48 hr), whereas the power exponential function was better suited for extended monitoring periods ranging from 49 to 219 days. Instead, we were able to highlight the robustness of fat‐tailed functions with data collected on short 6‐hr sessions; this indicates that a portion of the hooked diaspores could be dispersed over long distances.

The three ungulate vectors retained diaspores longer than our 6‐hr long experiment (16%, 26%, and 88% for dwarf goat, deer, and donkey, respectively). Poitou donkeys retained most of their diaspores due to their very long, thin, and flexible hair that easily tangles the diaspores and limits their dropping off (Couvreur et al., 2004, 2005). The dynamics of diaspore detachment were fairly similar for red deer and dwarf goat where nearly half of the diaspores fell off in the first 10 min, and then the remaining diaspores gradually fell off over the following hours, showing a very gentle slope. These dynamics are consistent with the findings of de Pablos and Peco (2007) who tested diaspore retention by shaking cow fur; they showed that poorly attached diaspores fell within the first few minutes whereas the proportion of better attached diaspores remained constant. The falling of the remaining diaspores seems therefore to be less related to animal movements than to other types of behavior. Nevertheless, after our 6‐hr monitoring sessions, the red deer still retained more diaspores in the fur than did dwarf goat. This result can be explained by the positive correlations revealed between diaspore retention capacity and hair length or fur depth. Dwarf goat has the shortest hair and the least thick fur of our three species. Such results also agree with experimental observations in Iberian ibex (*Capra pyrenaica*) highlighting that retention capacity of contact‐transmitted foreign particles was higher during the period in which ibexes had their longest hair coats (Sarasa et al., 2011). Further research could create a composite index of fur quality, taking into account hair density, length, thickness, and curliness (Albert, Mårell et al., 2015).

At the intraspecific scale (differences among body zones), the best functions varied from one animal species to another. The simple exponential function best fitted both dwarf goat and donkey and expressed a slow, gradual loss of the diaspores, while the power exponential function selected for red deer showed a faster loss of the first diaspores.

As expected and regardless of the animal vector, the diaspores attached to the head fell off more quickly. Hair is shorter on the head, and the fur and skin are thinner than on other body parts. Moreover, head movements are common to rid the animal of flies or humidity and are also linked to frequent social interactions and to mastication, even rumination. The flanks, for both red deer and dwarf goat, retained fewer diaspores than did the rump with longer and deeper hair. Graae (2002) made similar observations for dogs. In addition, compared to the rump, the flanks are more prone to regular contact with the environment, either by passing through branches, rubbing trees, or moving along fences. Diaspores attached to the head or the flanks of dwarf goat in our study were often recovered next to the fences around the pen and near trees.

Fur quality also varies across seasons. We would expect better retention capacity in fall/winter than in spring/summer fur and consequently shorter retention times and dispersal distances for diaspores moved in spring/summer than in fall/winter. Fall corresponds to the period of the year when most diaspores are released, but they may also remain attached to the dead twigs of the mother plant into the winter period. Once during our experiment with donkeys, we decided to leave the diaspores remaining in the fur at the end of the experiment for one more night. Checking the morning after revealed that not one single additional diaspore had disappeared. It would be interesting to regularly (once a day/week) monitor the fate of the attached diaspores until all of them had fallen off. In Poitou donkeys, diaspore retention time may extend until the loss of hair during the seasonal molt in the spring.

### 4.2 Grooming behavior

The presence of diaspores attached to their body seemed to affect the three ungulate species as they all demonstrated a tendency toward increased grooming, even though the trend was only significant for dwarf goat (stimulus‐driven‐grooming hypothesis). Our results also corroborate the body‐size principle based on the higher body surface to mass ratio found in the smaller ungulates like dwarf goat. Smaller body size probably means that large cocklebur diaspores are more irritating than for the other two larger ungulates, and dwarf goat therefore engaged more in auto‐grooming, thus nicely highlighting the parallel between diaspores and ectoparasites (Hart et al., 1992; Mooring et al., 2000). Dwarf goats groomed also more frequently in the absence of attached diaspores. We thus validated the second prediction of the body‐size principle stating that differences in programmed grooming rates persist even in the absence of ectoparasites, here in the absence of irritating diaspores. Although the percentage of fallen diaspores following grooming behavior did not significantly differ among species, grooming events were responsible of some diaspore detachment and may have been underestimated in our study. A significant percentage of fallen diaspores were found along fences and in the vicinity of trees; this is potentially due to voluntary rubbing. Diaspores could have been released near favorite rubbing trees and resting sites and this could be one mechanism of directed dispersal. Welander (2000) and Heinken, Schmidt, von Oheimb, Kriebitzsch, and Ellenberg (2006) both observed higher abundance and diversity of seeds next to trees where wild boar frequently rubbed.

### 4.3 Unsuspected case of diplochory: interindividual diaspore transfers

During our 6‐hr scanning sessions, we observed several real‐time interindividual diaspore transfer events (5% of the total amount of attached diaspores), for all three ungulate vector species. This happened only a few times for deer and dwarf goat, but significantly more for donkey (4 and 15 times more than for deer and dwarf goat, respectively). This is the first time that such observations have been reported to our knowledge. Donkeys are social animals that have multiple physical interactions among conspecifics. The fact that cocklebur diaspores tangle easily in donkey's long, thin hair explains why diaspore transfers were the main cause of diaspore losses. These diaspore transfers can be qualified as secondary seed dispersal events and are an unsuspected case of diplochory. These secondary dispersal events are undoubtedly underestimated and will complexify dispersal kernels as transferred diaspores might be transported to neighboring home ranges.

These transfers can be compared to ectoparasite transmission modes. Outputs of epidemiological models support the links between host density or local group size and the spread and diversity of directly transmitted parasites (Altizer et al., 2003; Anderson & May, 1979 and Arneberg, 2002). Monogamous species with strictly defended territories and which experience fewer intraspecific contacts should be less prone to ectoparasite infestations than social and gregarious mammals that live in multimale, multifemale, or fission/fusion groups (Altizer et al., 2003). The same could be true for diaspore transfers. Mammalian social systems result in temporal and spatial interactions among individuals. For instance, increased mounting behavior during the breeding season or increased contacts due to genetic relatedness has been observed, for example, between mother and juveniles (Alexander, 1974). Hausfater and Watson (1976) and Müller‐Graf, Collins, and Woolhouse (1996) have explained that parasitism is correlated with individual characteristics (dominance rank, age, sex, and mating status) which influence both habitat use and the frequency of intraspecific contacts. As social group size appears to predict parasite infestation risk in animals well (Côté & Poulin, 1995), we could hypothesize that vectors living in large social groups would correlate to long‐distance dispersal for epizoochorous plant populations.

### 4.4 Advantages and drawbacks of plant dispersal distance assessments for red deer

For our main experimental setup in RZHT, the dispersal distances were retrieved from hiking GPSs collared on our monitored red deer individuals. Each time a diaspore was lost, we were thus able to associate the precise distance covered by the hind. Contrary to the diaspore retention time function retained, the simple exponential function best fit our distance data. Fifty percent of the diaspores from the head and the flanks were released within the first 500 m, whereas rump‐diaspores fell off later on, after the animals had moved around 2 km. The main drawback encountered at the RZHT was that we monitored the dispersal process in an enclosure that ensured good visibility but restricted the animals in their movements (red deer hind's mean daily home range in situ of 81 ± 27 ha reported by Pellerin et al., 2016). That is why we presented the total distance covered by the animal from the beginning of the experiment until the fall of each attached diaspore to assess potential maximal dispersal distances instead of using biased Euclidian distances within the enclosure. The principal advantage at the RZHT was the possibility to directly observe diaspore detachment and its associated location.

For the classical approach where diaspore retention time functions were assessed under controlled conditions and combined with animal movements obtained in situ from telemetry data, there were also some disadvantages. The analysis of the data collected by the Hunting and Wildlife National Agency in February and March 2015 showed that the six equipped red deer individuals circulated on average within a radius of several hundred to several thousand meters between 9:00 and 15:00. The longest distances were covered during the first hours between 9:00 and 11:00, coinciding with the morning peak in activity observed for ungulates (Mooring et al., 2000). We were not able to tackle the specific dynamics of epizoochorous detachment as the 2‐hr interval between GPS locations neither matched the rapid loss of most of the diaspores nor allowed us to take path tortuosity into account (Pellerin et al., 2016).

Our innovative and direct approach to monitoring diaspore dispersal distance in situ has confirmed that most of the diaspores fell off during the first few minutes. We have also recovered one diaspore that became detached after 50 min 2.85 km from the animal's release site (Jégoux, 2016). Unfortunately, telephone signal reception away from the Chambord Castle was too weak and we therefore retrieved few, and only irregularly spaced, GPS locations via GSM to back track the released individuals. This limited our ability to recover detached diaspores in the field. A second drawback was linked to the animals’ capture: Tremendous stress occurred and the individuals bounded away from the release site with defense movements. Jeppesen (1987) showed that disturbed individuals moved several kilometers away, remained vigilant during the following hours, and then came back to their home range. The hunting season is a disturbing period for animals and lasts five months from the beginning of October until the end of February in the DNC, and such stressful situations like capture events might be much more common than expected. Jégoux (2016) studied the magnitude and timing of spatial reactions of red deer hinds in relation to hunting events in the DNC and found that they returned rapidly to their disturbance site, at the latest the day after. To improve this experimental approach, we could capture hinds via tele anesthesia to limit stress or we could test self‐attachment of diaspores from specific attraction sites (see for instance Sarasa et al., 2009). Also, we could select a site with better telephone coverage and increase the frequency of GPS acquisitions to one per minute. Different types of tracker may also be developed that store the location data and push the data to a server when the animal enters an area with mobile coverage.

The three approaches we implemented were complementary. They demonstrated the rapid loss of most diaspores and the opportunity for a few to be dispersed over much larger distances; we thus confirmed the potential of large native ungulates like red deer as long‐distance dispersal vectors for plants.

## CONFLICT OF INTEREST

None declared.

## AUTHORS’ CONTRIBUTIONS

CB formulated the idea. OL and CB contributed equally to this work. OL and CB designed the experiment. OL, MAG, CB, FJ, and FIN took part in the field work. OL performed data analysis. MAG performed GIS analysis. SS and CB imagined the innovative approach for dispersal distance assessment in situ. SS and FJ provided GPS data for red deer hinds. OL and CB wrote the manuscript. YL and FIN reviewed a final version of the manuscript. YL helped for the logistics in handling animals at RZHT.

## DATA ACCESSIBILITY

Analyses reported in this article can be reproduced using the raw data available from DRYAD: entry https://doi.org/10.5061/dryad.p1c3h.
